# INDIVIDUAL AND JOINT ASSOCIATIONS BETWEEN SLEEP AND PAIN ON MENTAL HEALTH: DIFFERENCES BY WORK STATUS AND OCCUPATION

**DOI:** 10.1093/geroni/igad104.1284

**Published:** 2023-12-21

**Authors:** Christina Mu, Soomi Lee

**Affiliations:** University of South Florida, Tampa, Florida, United States; The Pennsylvania State University, University Park, Pennsylvania, United States

## Abstract

Researchers have posited that healthcare workers (HCWs) are at greater risk of sleep and pain concerns due to their demanding work schedules and stressful workplace environment. Currently, more research is needed to understand the joint associations between sleep and pain and differences by work status and occupation type. This study examined the individual and joint associations between sleep and pain on mental health symptoms by workers (vs. non-workers) and HCWs (vs. non-HCWs). Data came from the Midlife in the United States (MIDUS) study (Time 1: 2004-2005; Time 2: 2013-2019). At baseline, 2,894 participants were working (2,640 were not working; n=294 HCWs). Sleep quality, sleep duration, number of pain locations, and pain interference were assessed. Over time, as sleep quality worsened, sleep duration shortened, and the number and interference of pain increased, then depressive and anxiety symptoms increased across all groups. The individual associations of poor sleep or more pain with depressive and anxiety symptoms were stronger among non-workers compared to workers. The relationships between poor sleep or more pain with depressive symptoms was stronger among HCWs compared to non-HCWs, but weaker or not statistically significant when anxiety symptoms was the outcome. There was evidence that the combination of poor sleep characteristics and greater pain exacerbated anxiety symptoms among all groups. Findings show differences in mental health as functions of sleep and pain by work status and HCW status. Evidence of the joint associations of sleep and pain support the interrelated nature of sleep and pain.

